# Reversal of a Fluorescent Fluoride Chemosensor from Turn-Off to Turn-On Based on Aggregation Induced Emission Properties

**DOI:** 10.1021/acssensors.1c02196

**Published:** 2022-01-12

**Authors:** Inmaculada Ortiz-Gómez, Sergio González-Alfaro, Antonio Sánchez-Ruiz, Ignacio de Orbe-Payá, Luís Fermín Capitán-Vallvey, Amparo Navarro, Alfonso Salinas-Castillo, Joaquín C. García-Martínez

**Affiliations:** † ECsens, Department of Analytical Chemistry, Faculty of Sciences, University of Granada, 18071 Granada, Spain; ‡ Unit of Excellence in Chemistry applied to Biomedicine and the Environment, University of Granada, 18071 Granada, Spain; § Universidad de Castilla-La Mancha, Departamento de Química Inorgánica, Orgánica y Bioquímica, Facultad de Farmacia, C/José María Sánchez Ibáñez s/n, 02008 Albacete, Spain; ∥ Universidad de Castilla-La Mancha, Regional Center for Biomedical Research (CRIB), C/Almansa 13, 02008 Albacete, Spain; ⊥ Department of Physical and Analytical Chemistry, Faculty of Experimental Sciences, Campus Las Lagunillas, Universidad de Jaén, 23071 Jaén, Spain

**Keywords:** Microfluidic paper-based analytical device, Fluorescent, Fluoride sensor, Aggregation Induced Emission, Chemosensor, DFT

## Abstract

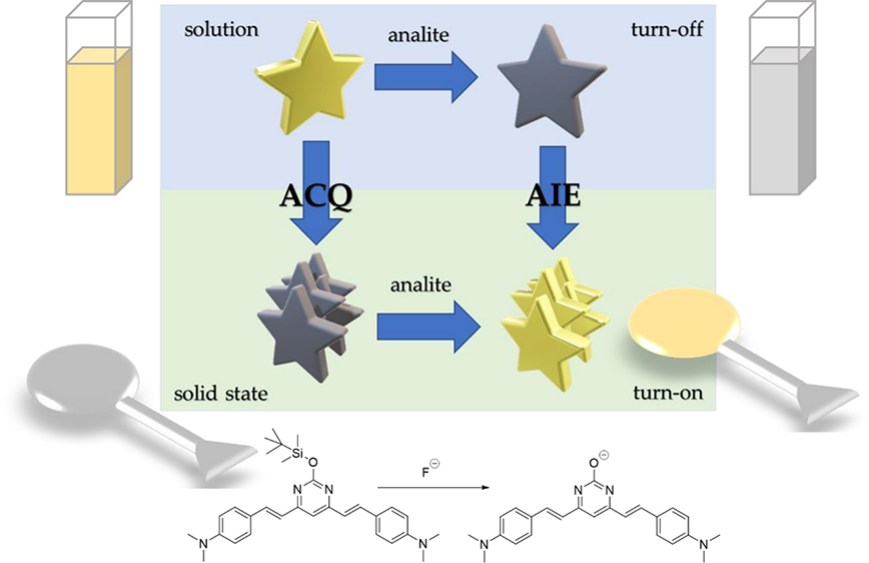

Here we present a new approach for the development of fluoride chemosensors taking advantage of aggregation induced emission (AIE) properties. Although AIE-based chemosensors have been described, they rely primarily on the analyte causing aggregation and hence fluorescence. We propose a new concept in the use of AIE for the development of fluorescent sensors. Our hypothesis is based on the fact that a turn-off chemosensor in solution can be transformed into turn-on in the solid state if the properties of ACQ and AIE are properly combined between the fluorescent molecules involved. To demonstrate this hypothesis, we have selected a fluorescent chemosensor for the fluoride anion with a conjugated structure of bis(styryl)pyrimidine that, while showing turn-off behavior in solution, becomes turn-on when it is brought to the solid state. We have also combined it with the advantages of a detection system based on the microfluidic paper-based analytical devices (μPAD). The system is fully characterized spectroscopically both in solution and in the solid state, and quantum mechanical calculations were performed to explain how the sensor works. The prepared device presents a high sensitivity, with no interference and with an LoD and LoQ that allow determination of fluoride concentrations in water 2 orders of magnitude below the maximum allowed by WHO.

Aggregation Induced Emission is a phenomenon coined as AIE in 2001^1,2^ which has come to the attention of the scientific community. For a large number of nonplanar molecules, this phenomenon could be attributed to the blocking of rotational and vibrational motions of the molecule when going from solution to the solid state through the so-called restriction of the intramolecular motions (RIM) mechanism. However, more complex inter- and intramolecular processes can also be engaged, such as *cis–trans* photoisomerizations, short and long-range excitonic coupling interactions, or restricted access to conical intersection in the excited state (RACI), among others.^3,4^ As expected, this phenomenon aroused the interest of many researchers and a huge number of fluorescent chemosensors based on AIEgen^5,6^ (molecules with AIE properties) have been described for the detection of cations,^7^ anions,^8^ neutral molecules, and biomolecules.^9,10^ The detection mechanisms employed in all these cases can be summarized in three ways, as shown in Scheme 1 (i): (a) the analyte induces aggregation of the AIEgen leading to an increase in fluorescence, giving a so-called turn-on sensor. This may occur because the analyte induces the self-assembly of the AIEgen or because the analyte reacts yielding a more insoluble and aggregating AIEgen. (b) The analyte induces the disaggregation of the AIEgen leading to a loss of fluorescence and a turn-off type sensor. (c) The analyte induces a modification in the chemical structure of the AIEgen resulting in different optical properties, thus providing a ratiometric type sensor. The aggregation is usually done in solution in almost all cases, and the most common solvent is water due to the hydrophobic nature of the AIEgens, although there are few examples where such sensors have been brought to solid state. Two examples are described by Tang et al.^11^ and Jiang et al.,^12^ in which sensors for amine vapors and cyanide in water are described, respectively. Both are based on the fact that a hydrogen bond is formed, which is responsible for an excited-state intramolecular proton transfer (ESIPT) that produces a restriction of intramolecular motion mechanism (RIM). The result is an enhancement of the fluorescence emission. In both cases, the sensor works the same way in solution as in the solid state.

**Scheme 1:**
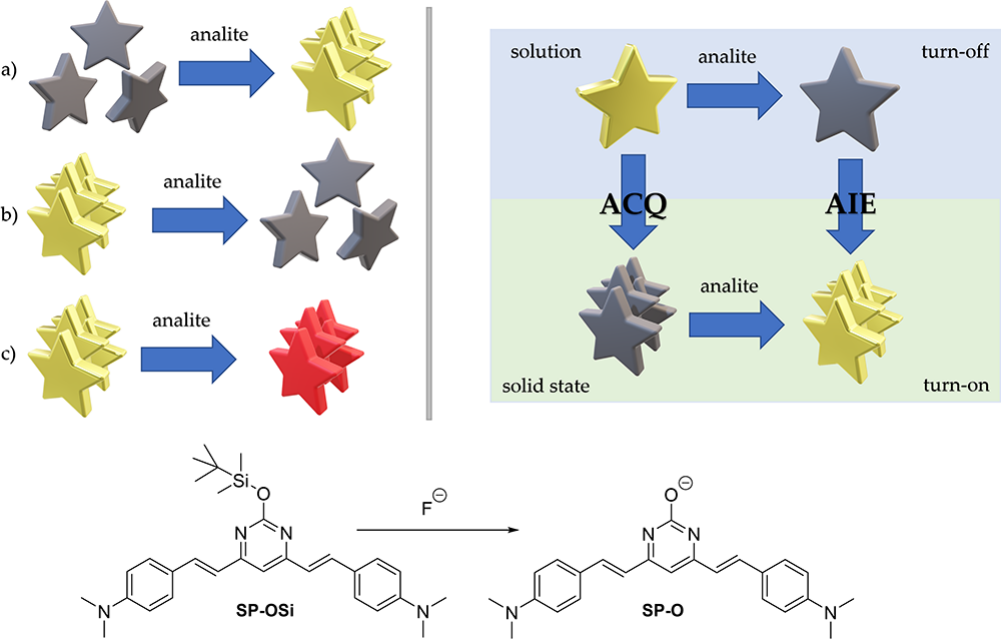
(i) Types of Fluorescent Chemosensor Based on AIE. (ii) Schematic Representation of How a Turn-Off Chemosensor in Solution May Become Turn-On in Solid State. (iii) Sensor Reaction for the Determination of Fluoride Gray color of the stars indicates non-emissive compounds, gold color indicates fluorescent compounds, and red color indicates change in the emission wavelength of the compound.

Here, we present a new concept for the development of fluorescent chemosensors based on solid-state AIE (Scheme 1(ii)). It is based on the idea that classical turn-off chemosensor systems in solution can be converted into turn-on systems in the solid state if the two species involved, the reagent and the product, exhibit ACQ and AIE, respectively; that is, if the reagent presents ACQ, it would lose fluorescence in the solid state, while if the product presents AIE, it will fluoresce in solid state. More specifically, in a turn-off chemosensor in solution, the reactive species is emissive but loses its fluorescence when it reacts with the analyte to give the nonemissive product. Furthermore, if the same reaction is carried out in the solid state, it would lead to a turn-on type sensor. This four-points mechanism would open the door to reconsidering chemosensors that have not been of great interest because of their turn-off behavior in solution, but that could be salvaged by moving to the solid state.

An example of these are fluoride sensors, whose importance arises from the fact that this anion plays a significant role in fields as diverse as health, chemical weapons manufacture, or uranium refinement.^13,14^ Although fluoride chemosensors with good detection levels in organic solvents have been described,^15−17^ the use of water as solvent limits their applications because of the low solubility of fluorophores and, above all, because of the high degree of solvation of fluoride in water, which greatly reduces its reactivity. For this reason, we believe that the four-point sensor that we present here could be an ideal candidate to demonstrate our methodology and transform a turn-off sensor in solution into a turn-on sensor in the solid state on aqueous samples. AIE-based fluoride chemosensors have been described previously, all based on the induction of AIEgen aggregation or disaggregation,^18−20^ disruption of conjugation,^21^ or induction of an ESPIT process.^22^

In order to implement the fundamentals of our idea, we are going take advantage of the microfluidic paper-based analytical devices (μPAD).^23−25^ μPAD has become a common and powerful design tool for the fabrication of sensors and biosensors, because of its inherent properties such as flexibility, low cost, light weight, tailorability, environmental friendliness, degradability, and renewability. In addition, it presents singular properties because its intrinsic naturally porous structure, a physical characteristic that enables paper for spontaneous capillary-based fluidic transport of sample and reagents, also confers the benefits of reagent storage, mixing, flow control, and multiplex analysis. In addition, paper substrate provides a solid platform with attractive properties such as a porous structure, capillary action driven sample flow, flexibility, ease of modification, and availability. Currently, the use of microfluidic paper-based analytical devices has shown high potential to be applied in a variety of food and environmental analysis, sensing, and diagnostic applications.

Here, we present a fluorescent chemosensor for the fluoride anion with a conjugated structure of bis(styryl)pyrimidine that, while showing a turn-off behavior in solution, becomes turn-on when it is brought to the solid state. It is based on the cleavage of a Si–O bond by reaction with fluoride to form the corresponding pyrimidinolate (Scheme 1(iii)). A detailed theoretical and experimental study of the optical properties of both species is shown here with the purpose of explaining the spectroscopical phenomena that occurs, as well as the results as a fluoride sensor in solution. The sensor is brought to the solid state in a μPAD, and after full characterization, a comprehensive analysis of the fluoride assay on paper is presented.

## Results and Discussion

The preparation of the bis(styryl)pyrimidine **SP-OSi** was achieved by condensation of the 4,6-dimethylpyrimidin-2-ol and 4-(dimethylamino)benzaldehyde in acidic media followed by further treatment with *tert*-butyldimethylsilyl chloride, leading to the final compound **SP-OSi**. All the details of synthesis and characterization of the compound are given in the Supporting Information along with computational details of the theoretical calculations. Before going into the details of the sensor, the reaction with fluoride was confirmed by ^1^H NMR and HR-MS (Figures S1 and S2). Comparison of the NMR spectra of both compounds, **SP-OSi** and **SP-O**, revealed that all aromatic and olefinic signals exhibited lower chemical shifts in **SP-O** than in **SP-OSi**, as expected from the presence of an oxyanion in the structure. For instance, pyrimidine proton was the most affected one, as it moved from 6.78 ppm in **SP-OSi** to 6.15 ppm in **SP-O**, due to the strong electron-donating character of the oxyanion. In the case of mass spectrometry, when fluoride is added, the signal at 501.3053 Da disappears immediately after reaction and the signal corresponding to **SP-O** anion appears. In the sweep of the spectrum, the adducts corresponding to the M+H, M+Li, and M+Na are observed (Figure S2).

The first step to address this study was the optical characterization of both the silylated **SP-OSi** sensing molecule and the final product obtained by breaking the O–Si bond, **SP-O**. The latter was obtained by treatment of the **SP-OSi** compounds with an excess of KF aqueous solution. The experimental UV–vis absorption and emission spectra were obtained in different organic solvents, and the results are summarized in Table 1 and Figure S3 in the Supporting Information. **SP-OSi** consists of a pyrimidine ring with electron withdrawing character and two dimethylamino-substituted styryl branches with electron-donating character. Among the most noteworthy aspects are that the combination of donor and acceptor groups in the structure gives rise to a dependence of the optical properties on the polarity of the solvent as indicated in the Lippert–Mataga diagram (see Figure S4a in SI). The fluorescence quantum yield was also affected by the polarity of the solvent and decreased as the polarity increased. Thus, the **SP-O** showed a Φ = 39% in toluene, whereas in acetonitrile it dropped to 5%. The dependence of the emission with the polarity of the solvent was more pronounced in the final product after fluoride treatment where the corresponding hydroxypyrimidine anion was generated. In this case, the slope of the Lippert–Mataga diagram was greater than for the neutral compound. The quantum yields of the anionic **SP-O** species also decreased with solvent polarity, always being lower than the silylated (**SP-OSi**) precursor compounds for the same solvent (toluene Φ = 19%, acetonitrile Φ = 8%). Additionally, solvent–nonsolvent experiments with DMSO/water mixtures were carried out to induce aggregation in both species (Figure S4b). As the fractions become water-enriched, **SP-OSi** decreases the quantum yield, while **SP-O** increases the fluorescence before dropping by precipitation of the compounds, as expected for ACQ and AIE effect, respectively (*vide infra*).

**Table 1:** Spectroscopic Data of **SP-OSi** and **SP-O** in Different Organic Solvents^a^

compound	solvent	λ_ab_^max^ (nm)	ε^b^ (l mol^–1^ cm^–1^)	λ_em_^max^ (nm)	Φ
SP-OSi	Tol	459	36.2	549	39
	THF	435	41.3	557	33
	ACN	469	20.5	631	5
	DMSO	468	36.7	617	7
	Thin film	404^c^	-	480	0
	μPAD	445^c^	-	565	3
SP-O	Tol	438, 313	29	538	19
	THF	404, 355	31.6	510	11
	ACN	407, 350	47.9	467	8
	DMSO	413, 358	37.2	467	2
	Thin film	410^c^	-	482	7
	μPAD	520^c^	-	608	33

a Maximum absorption energy (λ_ab_^max^), molar absorption coefficients (ε), maximum emission energy (λ_em_^max^), Stokes shift and quantum yield (Φ) determined in various solvents (Tol, THF, ACN, and DMSO correspond with toluene, tetrahydrofuran, acetonitrile, and dimethyl sulfoxide, respectively).

b ×10^3^.

c In solids, the maxima correspond to the maxima of the excitation spectrum (λ_ex_^max^).

The properties of both compounds were studied in the solid state, either drop-cast on a quartz disc or on a disc of Whatman no. 1 chromatography paper. In both cases, a few drops of solution of the corresponding compound were placed on the corresponding surface and the solvent was allowed to evaporate. The most interesting fluoride samples were aqueous, and the high degree of solvation of fluoride anions meant that many of the sensitive molecules developed for this anion gave very low sensitivities in water. We have recently demonstrated how, by dissolving the sensor in DMSO, it is possible to use aqueous fluoride samples with excellent sensitivities.^15^

**Figure 1:**
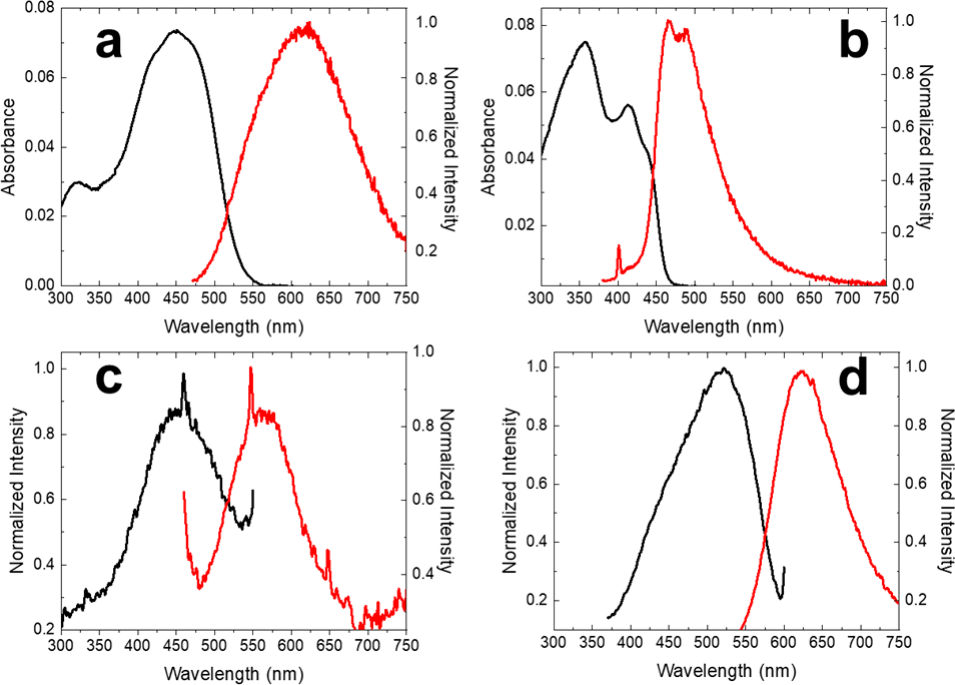
UV–vis and fluorescence spectra in 2 μM of DMSO of **SP-OSi** (a) and **SP-O** (b). Excitation and fluorescence spectra in μPAD of **SP-OSi** (c) and **SP-O** (d).

For this reason, Table 1 includes the results obtained for both compounds in DMSO solution, along with on a quartz disc and on a paper disc. The silylated compound **SP-OSi** showed a hypsochromic shift of absorbance and emission from DMSO solution to solid (thin film and μPAD), as well as a decrease of the quantum yield in the emission spectrum (Figure 1 and Table 1). Both effects, hypsochromic shift and quenching of the emission (ACQ effect), were associated with the formation of H aggregates in the solid state in the context of Kasha’s theory. On the contrary, the **SP-O** anion showed a bathochromic shift of the absorbance and the emission from DMSO solution to μPAD paper (Figure 1 and Table 1) along with an increase of the emission intensity which could be attributed to the formation of J-type aggregates in the context of Kasha’s theory, thus resulting in AIE effect. The high permanent dipole moment predicted for **SP-O** (see Figure S6) could promote electrostatic repulsions between anionic charges and dipole–dipole interactions, which strongly influenced specific organization of molecules in the solid state and, consequently, the emission-yielding emissive aggregates. However, the **SP-OSi** is a neutral species with lower dipole moment and planar molecular scaffold, which could promote π–π intermolecular interactions that quench the emission in the solid state in a typical H-type aggregation.

The decrease of the quantum yield in DMSO solution from **SP-OSi** to **SP-O** was rationalized by performing TD-DFT calculations at the TD-M06-2X/6-31+G** level of theory. The molecular geometry for the ground and first excited state of both molecules was optimized in DMSO solution with some selected geometrical parameters are shown in Figure S6. In both cases, the central skeleton shows planar geometry with dihedral angles smaller than 1.4° which were slightly diminished after excitation. However, the dimethylamino moiety shows certain pyramidal structure in the ground state with C–C–N–C dihedral angles around 7° in the case of **SP-OSi** and 11° for the **SP-O**. Upon excitation, the two dimethylamino groups in **SP-OSi** are planarized, reaching dihedral angles of 0.1°, while in the case of the **SP-O**, only one dimethylamino group is planarized (0.2°), while the other one remains in an 11° dihedral. The changes in the planarity of the electron-donating dimethylamino group after excitation directly impacts the intramolecular charge transfer from the periphery to the central part of the molecular skeleton. This effect could produce different locally excited (LE) and twisted intramolecular charge-transfer (TICT) states responsible for the emission^23^ with their relative stability depending on the polarity of the solvent.^24,26^ Therefore, we postulate that in nonpolar solvent the emission would occur from the LE state resulting in high quantum yields in solution (see Table 1). However, in polar solvent as acetonitrile and DMSO, the TICT state would become more stable yielding a nonradiative relaxation and a low quantum yield in solution. This effect should be more pronounced in the case of the anion, and for this reason, it shows lower quantum yield than the neutral form. A thorough study of these effects will be the subject of future work.^27^

The vertical electronic transitions were calculated for the neutral and anionic compounds (see Table S1). The lowest energy transition S_0_ → S_1_ is predicted at 397 nm for **SP-OSi** with a high oscillator strength of 2.17 and a large contribution of HOMO → LUMO transition (86%). In the case of the **SP-O** anionic compound, the S_0_ → S_1_ transition is predicted at 374 nm with oscillator strength of 1.74 and a large contribution of HOMO → LUMO transition (81%). These theoretical results are in good agreement with the experimental observations showing that the main electronic transition S_0_ → S_1_ presents intramolecular charge transfer character. The HOMO and LUMO molecular orbitals are shown in Figure S7. For the neutral molecule, the HOMO is delocalized over the vinylene arms and the dimethylamino groups, while the LUMO is located in the central pyrimidine ring. In the case of anion, the HOMO is delocalized over the whole of the molecule.

**Figure 2:**
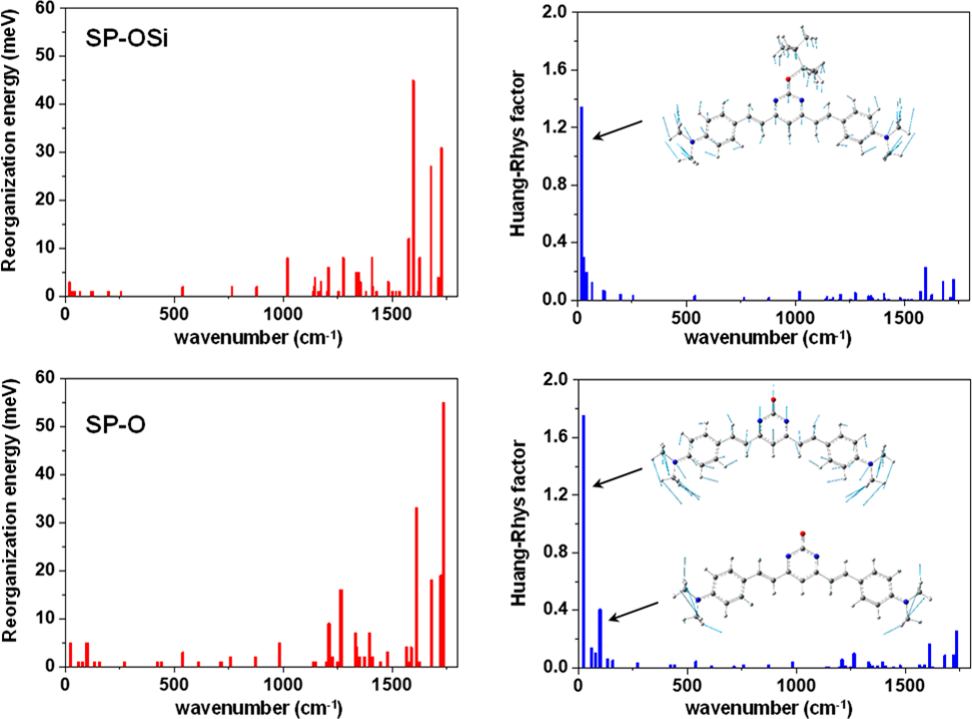
Reorganization energy and Huang–Rhys factor versus normal mode wavenumber of the ground state for compounds **SP-OSi** and **SP-O** in DMSO computed at the at the M06-2X/6-31+G** level of theory.

The fluorescence emission was also calculated for the first excited state in DMSO solution obtaining a reasonable agreement with the experimental data (see Tables 1 and S1). In order to probe more deeply into the decrease of the fluorescence quantum yield from **SP-OSi** to **SP-O**, the reorganization energy and Huang-Rhys (HR) factors were calculated which account for the nonradiative vibrational relaxation from the excited state (see Figure 2 and Table S2). As shown in Figure 2, there are two normal modes at 18 and 23 cm^–1^ with a significant HR factor of 1.34 and 1.75 for **SP-OSi** and **SP-O**, respectively. The higher value in the case of the anionic species is in agreement with the decrease of the quantum yield from 7% in **SP-OSi** to 2% for **SP-O** due to the most favored vibrational relaxation. In summary, the lower emission of **SP-O** compared to **SP-OSi** in solution could be due to the higher HR factors calculated in the first case which are associated with the vibrational nonradiative relaxation from the excited state. However, when the **SP-O** compound is in the solid state, these vibrational motions are restricted, leading to an increase of the fluorescence quantum yield from solution to solid state. Additionally, and as deduced from the UV absorptions, **SP-OSi** could stack giving rise to H-type aggregates that lead to a quenching of fluorescence, while **SP-O** does by means of J-type aggregates leading an emissive compound in the solid state. In addition, the intramolecular torsion of the amine group in the **SP-O** would be restricted in μPAD and then would disfavor the nonradiative relaxation from the TICT state, resulting in bright state after aggregation.^28,29^

In general, fluorescent fluoride sensors have the major drawback of low sensitivity when water is used as a solvent, and this is a major problem when the samples and sources of interest for detecting fluorides are aqueous, such as rivers, lakes, wastewater, tap water, etc. We have recently developed a methodology in which by dissolving the sensor molecule in DMSO it is possible to add the fluoride analyte in water and obtain very good sensitivities.^15^

We have used this methodology to study the behavior of this fluoride sensor in solution. Thus, the sensitivity toward fluoride was investigated by adding increasing amounts of KF to 2 μM solutions of **SP-OSi** in DMSO. KF causes the cleavage of the Si–O bond and the formation of the **SP-O** compound. As **SP-OSi** fluoresces more strongly than **SP-O**, the result is a net decrease on fluorescence as the concentration of fluoride increased in a typical turn-off sensor behavior. The data points were then fitted to a linear function (Figure S8 of the Supporting Information), and the linear range was found to lie between 4.2 μM and 158 μM after its calculation using the method described by Sebaugh and McCray.^30^ In order to rule out any effect produced by water and not by fluoride, we examined how the fluorescence evolved with different amounts of water (Figure S9). It was observed that the fluorescence remained constant even at amounts seven times higher than those added in the calibration experiments. Furthermore, to confirm that the basic character of the pyrimidine also has no effect on the sensor, titration in HCl was also performed, observing that at pH above 6, the fluorescence remains constant (Figure S9).

The limits of detection (LoD) and quantitation (LoQ) were determined to be 14.5 μM and 48.3 μM, respectively. These values are similar to other fluorescent fluoride sensors^15^ and below the maximum permissible drinking water contamination level of 4 ppm (210 μM).^31^ The selectivity was studied in an initial screening by exposing **SP-OSi** to excess of different salts (1 mM final concentration for each salt), such as KCl, KBr, tetrabutylammonium chloride (TBAC), tetrabutylammonium bromide (TBAB), K_2_CO_3_, KNO_3_, MgSO_4_, KOH, NaCN, and sodium acetate (AcONa). The overall fluorescence intensities were not affected significantly, except for KOH, where it was competitive with fluoride (Figures S10 and S11). This effect was probably due to the lability of the O–Si bond at highly basic pH values. In general, the useful samples of interest do not usually have high pHs; however, buffered solutions can be used to eliminate this issue.

**Figure 3:**
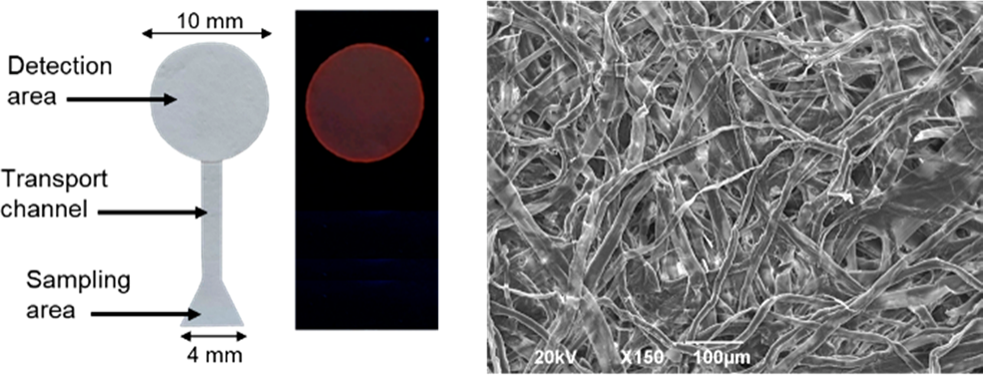
On the left, picture of the μPAD device showing sizes and the two layers: detection area, transport channel, and sampling area and picture under UV light after fluoride ion detection. On the right, SEM photograph of the detection area after **SP-OSi** loading.

The μPAD device was prepared according to our experience,^32^ and the details are described in the SI. Figure 3 shows a picture of the μPAD with the elements and dimensions. The 10 μM THF solution of **SP-OSi** was deposited on the detection disk and allowed to wet the paper before it fully evaporated. The aqueous fluoride sample under study was placed in the sampling area and allowed to reach the detection area through capillarity. Figure 3 and Figure S12 shows pictures of the disk emitting light under UV light before and after treatment with fluoride. Figure 3 on the right and Figure S13 in SI show SEM photographs of the paper before impregnation with **SP-OSi**, after loading, and after fluoride treatment. The highly conjugated structure of the molecule allows visual detection. To confirm the presence of the molecule on the paper, EDS analysis was carried out, showing the presence of Si in the sample loaded with **SP-OSi** and F in the one treated with fluoride (Figure S14 in SI).

**Figure 4:**
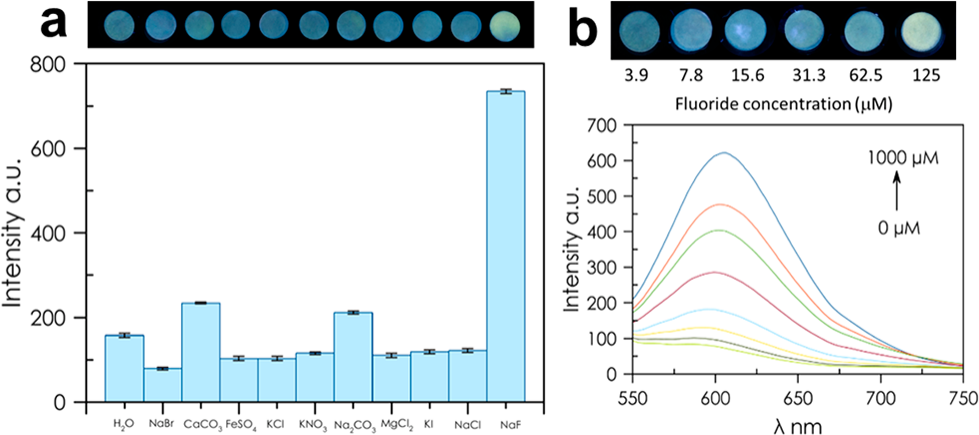
(a) Selectivity toward different anions in μPAD system and picture of μPADs under UV lamp irradiation (365 nm) after incubation with different anions. (b) Emission spectral response of **SP-OSi** with increasing concentrations of fluoride ions and picture of μPADs used to build the calibration.

In order to ensure the best performance of the μPAD for the evaluation of fluoride ions, different variables involved in the engineered steps were optimized such as (a) concentration of **SP-OSi**; (b) pH; (c) volume of sample; and (d) reaction time (Figure S15). The following experimental conditions were found to give best results: (a) The reagent **SP-OSi** was deposited on the detection area of the μPAD from a THF solution because of its high solubility and evaporation rate in this solvent. The optimum **SP-OSi** amount immobilized was studied by drop casting 4 μL per detection area of a solution with concentrations ranging between 2.5 and 20 μM; the optimal concentration was found to be 10 μM. (b) **SP-OSi** presents a strong stability at in the pH range 6–8. (c) Different volumes (5–20 μL) of sample solution were added onto the sampling area of the μPAD, with 20 μL being the optimal sample volume, as it is enough to wet the μPAD with a reasonable drying time and good precision. (d) Reaction time was studied by measuring the detection area of the μPAD at different times, with 20 min being the best balance between completion of the reaction and full evaporation of the aqueous solution. Figures 4b and S16 shows how the fluorescence in the μPAD increases as the fluoride concentration increases and how this relationship is linear allowing a calibration curve. Its selectivity was evaluated using NaBr, CaCO_3_, FeSO_4_, KCl, KNO_3_, Na_2_CO_3_, MgCl, KI, KOH, and NaCl as potential interfering ions with or without NaF as the source of fluoride present, and μPAD after incubation with mixtures of 20 μM NaF in the presence of 20 μM of different ions. The data presented in Figures 4a and S10 show that **SP-OSi** exhibited excellent selectivity as a fluorescence probe for fluoride ions over other inorganic salts, either with each salt alone or combined with fluoride.

Finally, the μPAD system was tested with real-life samples to investigate the practicality of the developed method; it was employed to detect fluoride ions in tap water, green tea, and two commercial products containing fluoride in their composition: mouthwash and toothpaste. Tap water and green tea were measured directly with the device, while commercial mouthwash and toothpaste required a pretreatment which is described in the SI. The results are shown in Table 2; the fluoride concentration was measured to be 2.5, 1.1, 218.1, and 246.2 mg/L, respectively. Furthermore, spiked recovery experiments were carried out and the recoveries were between 108.2% and 115.6%, confirming the suitability of this approach for the analysis of fluoride ion in real samples.

**Table 2:** Determination of Fluoride Ions in Real Samples (*n* = 3)

sample	fluoride found	added μM	found μM	recovery %
Tap water	60.2 ± 1.7^a^	20.0	87.9	109.7
		60.0	138.8	115.6
Green tea	28.0 ± 1.2^a^	20.0	54.4	113.0
		60.0	95.3	108.2
Mouthwash	218.11 ± 1.8^b^			
		
Toothpaste	246.2 ± 1.9^b^			

a Concentration in μM.

b Concentration in ppm. Mouthwash sample labeled 220 ppm. Toothpaste sample labeled 250 ppm.

## Conclusions

We have described an innovative analytical approach for the detection of fluoride anion on a fluoride chemosensor containing a 2-*tert*-butyldimethylsilyloxypyrimidine scaffold as the electron accepting moiety and 4-dimethylaminostyrene as the electron donating group. Its initial (**SP-OSi**) and final (**SP-O**) forms exhibit ACQ and AIE in solid state, respectively. This effect has been analyzed through quantum mechanical calculations, which revealed that in solution the drop in fluorescence emission from **SP-OSi** to **SP-O** is due to the higher nonradiative vibrational relaxation of the latter species. However, when the sensor is deposited in μPAD, an increase in the emission after the analysis reaction is produced probably due to two factors, a J-type stacking and the fact that the nonradiative vibrational relaxation becomes restricted. Therefore, the combination of one species with ACQ (**SP-OSi**) and another with AIE (**SP-O**) allows reversal of a turn-off type sensor in solution into a turn-on in the solid state. This effect was taken advantage of to develop an inexpensive and easily built μPAD system, which could detect fluoride with a 10-fold improvement in sensitivity over the same chemosensor in solution and with similar selectivity toward fluoride in the presence of other ions. To the best of our knowledge, this is the first example in the literature in which, by taking advantage of the AIE properties of the molecule, a turn-off sensor in solution is transformed into a turn-on sensor in the solid state, μPAD. This is an important milestone, as it means that many turn-off fluorescent chemosensors in solution could be transformed into a turn-on one when deposited in the solid state on a paper device, if the spectroscopic properties so dictate.

## Supporting Information Available

Experimental section, synthetic procedures, characterization, equipment used, and computational details. (PDF)
