# Supplementary material for: Reversal of a Fluorescent Fluoride Chemosensor from Turn-Off to Turn-On Based on Aggregation Induced Emission Properties
Source: ACS Sens. 2022 Jan 12;7(1):37–43. doi: 10.1021/acssensors.1c02196 (PMC8805153; doi:10.1021/acssensors.1c02196)
Supplement: Supplementary file 1 — se1c02196_si_001.pdf [file se1c02196_si_001.pdf]

Reversing of a Fluorescent Fluoride Chemosensor from Turn-off to Turn-on Based on Aggregation Induced Emission Properties

Inmaculada Ortiz-Gómez[1,2#], Sergio González-Alfaro [3,4#], Antonio Sánchez-Ruiz [3,4], Ignacio de Orbe-Payá[1,2], Luís Fermín Capitán-Vallvey[1,2], Amparo Navarro[5], Alfonso Salinas-Castillo[1,2*], Joaquín C. García-Martínez[3,4*]

[1] *ECsens, Department of Analytical Chemistry, Faculty of Sciences, 18071 University of Granada, Granada, Spain.*

[2] *Unit of Excellence in Chemistry applied to Biomedicine and the Environment, 18071 University of Granada, Granada, Spain.*

[3] *Universidad de Castilla-La Mancha, Departamento de Química Inorgánica, Orgánica y Bioquímica, Facultad de Farmacia, C/ José María Sánchez Ibáñez s/n, 02008, Albacete, Spain.*

[4] *Universidad de Castilla-La Mancha, Regional Center for Biomedical Research (CRIB), C/ Almansa 13, 02008, Albacete, Spain.*

[5] *Department of Physical and Analytical Chemistry, Faculty of Experimental Sciences, Campus Las Lagunillas, 23071 Universidad de Jaén, Spain.*

[*]Corresponding authors: E-mail address: JoaquinC.Garcia@uclm.es (Joaquin C. García-Martínez); alfonsos@ugr.es (Alfonso Salinas-Castillo).

[#]Contributed equally

ORCID:

Inmaculada Ortiz-Gomez: 0000-0003-4677-2383

Ignacio Orbe-Payá: 0000-0003-2308-6241

Luis Fermin Capitan-Vallvey: 0000-0002-7112-3601

Sergio González-Alfaro: 0000-0002-1937-7401

Antonio Sánchez-Ruiz: 0000-0002-3656-7442

Amparo Navarro: 0000-0001-9620-6668

Joaquín C. García-Martínez: 0000-0003-3321-4243

Alfonso Salinas-Castillo: 0000-0002-1360-6699

KEYWORDS: Microfluidic paper-based analytical device · Fluorescent · Fluoride determination

1. Materials and methods

1.1. Materials and reagents

Tetrahydrofuran (THF), dimethyl sulfoxide (DMSO) potassium hydroxide, potassium fluoride, sodium fluoride (NaF), potassium iodide, sodium chloride, sodium bromide, potassium chloride, magnesium chloride, potassium nitrate, sodium carbonate, calcium carbonate, ferric sulphate (II), were purchased from Sigma Aldrich. Whatman no 1 chromatography paper from Sigma-Aldrich was used to prepare the microfluidic paper-based analytical device (μPAD). Aqueous solutions were all prepared using reverse-osmosis type quality water (Milli-RO 12 plus Milli-Q station from Millipore, conductivity 18.2 MΩ·cm).

1.2. Apparatus, instruments and software

^{1}H NMR and ^{13}C NMR spectra were acquired at room temperature in VARIAN 400MHz. The chemical shifts (δ) are given in ppm and are referenced to the residual protons of the deuterated solvent or carbon nuclei of chloroform (^{1}H, δ= 7.27 ppm; ^{13}C, δ= 77.0 ppm) or DMSO (^{1}H, δ= 2.50 ppm; ^{13}C, δ = 39.5 ppm). Acidic impurities in $CDCl_3$ were removed by treatment with anhydrous K_2CO_3. IR spectra were recorded on an FT-IR spectrophotometer equipped with an ATR accessory and the main peaks are given in cm^{-1}. MALDI-TOF mass spectrometry was carried out on an Autoflex maX (Bruker) and high-resolution mass spectrometry (HR-MS) was carries out on an LCT Premier XE.

The μPAD were fabricated using a craft-cutting technique by cutting the paper using a 12W CO_2 laser engraver (Rayjet, Barcerlona, Spain). Fluorescence spectrums were collected using a Varian Cary Eclipse luminescence spectrometer (Varian Ibérica, Madrid, Spain). For μPAD samples, excitation spectra were recorded on a Varian Cary Eclipse fluorescence spectrophotometer in the range 550 – 800 nm with a xenon lamp as excitation source. Luminescence intensity was measured using the following conditions: λ_{ex} = 530 nm and λ_{em} = 617 nm, the photomultiplier voltage was set at 550 V and excitation and emission slits of 5 nm were used.

UV-Vis absorption spectra were acquired on a Cary 100 (Varian) spectrophotometer at room temperature using a slit width of 0.4 nm and scan rate of 600 nm/min. Steady state fluorescence were recorded on an FLS920 system (Edinburgh Instruments) equipped with a time correlated single photon counting (TCSPC) detector. A TLC 50 temperature-controlled cuvette holder (Quantum Northwest) was used for the measurements (temperature was controlled at 296

K). Quartz cuvettes (Hellma Analytics) of 10 mm were employed for all the spectroscopic measurements. For SSF spectra, a Xe lamp of 450 W was used as the light source and the excitation and emission slits were both fixed at 1 nm. The step and dwell time were 1 nm and 0.1 s, respectively. The quantum yields of the compounds in solution and solid state were recorded on an FS5 system (Edinburgh Instruments) equipped with a time-correlated single-photon counting (TCSPC) detector and an integrating sphere module (SC-30). The quantum yields of solutions were measured using 10 mm quartz cuvettes (Hellma Analytics). In solid, the quantum yields were obtained from the corresponding drop cast samples over quartz slides and directly from the μPad paper and measuring both the direct and the indirect fluorescence emission. In all cases, the step and dwell time were 1 nm and 0.3 s, respectively.

The microstructure was evaluated by Scanning Electron Microscopy (SEM) using a Jeol 6490LV electron microscope equipped with SE and BSE detectors and an Oxford Link EDS Probe. The samples were prepared by placing a spatula tip of the organogel on top of a sampler stub and allowing them to dry for 6 h at room temperature. Specimens for SEM observation required coating (Au-Pt, Emitech) to avoid problems related to the surface charging-up.

2. Synthesis of SP-OSi

Compounds **1** and **3** were prepared according to literature[1] and compound **4** was prepared as follows:

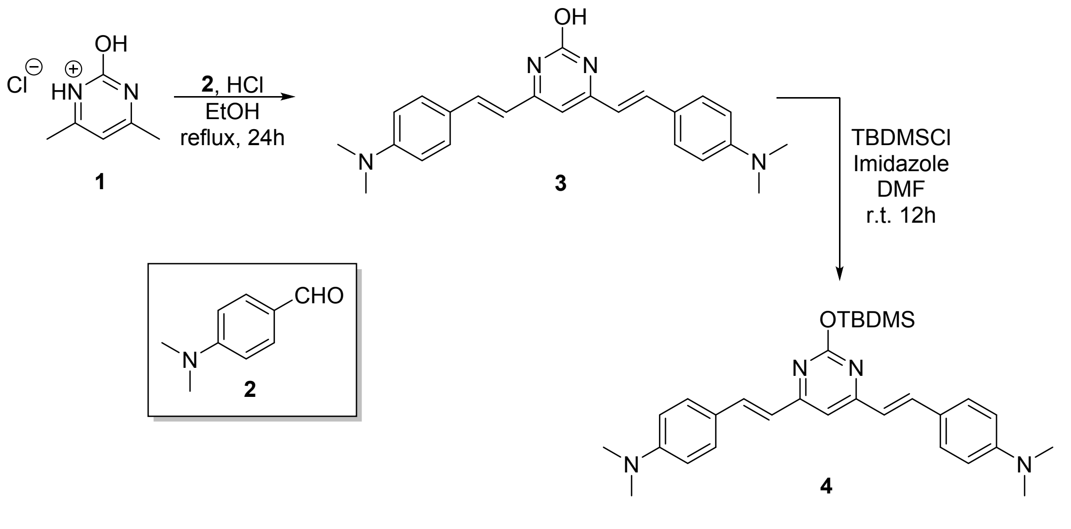

Scheme S1. Synthesis of SP-OSi **4**.

[1] Liu, B.; Zhang, H.; Zhao, Y.; Luo, Q.; Huang, Z. Novel pyrimidine-based amphiphilic molecules: synthesis, spectroscopic properties and applications in two-photon fluorescence microscopic imaging *J. Mater. Chem.* **2007**, *17*, 2921-2929

Compound 3 (166 mg, 0.43 mmoles, 1.0 eq.) was dissolved in DMF (10 mL) and imidazole (188 mg, 2.76 mmole, 6.4 eq.) and TBDMSCl (354 mg, 2.36 mmoles, 5.5 eq) were sequentially added, and the reaction was stirred at room temperature for 14h. After this time, TLC (AcOEt 50% in hexanes) showed completion of the reaction. Mixture was diluted with NH_4Cl (satd) and extracted twice with Et_2O. The organic phase was dried (MgSO4), evaporated and the crude purified through column chromatography (AcOEt 25% in hexanes) to yield 142 mg of BDMASP-OSi 4 (66%). Rf (AcOEt 50% in hexanes with 1% Et_3N): 0.79. ^{1}H-NMR (500 MHz, $CDCl_3$) δ 7.75 (d, *J*= 15.8 Hz, 2H), 7.48 (d, *J*= 8.9 Hz, 4H), 6.82 (d, *J*= 15.8 Hz, 2H), 6.78 (s, 1H), 6.70 (d, *J*= 8.9 Hz, 4H), 3.01 (s, 12H), 1.07 (s, 9H), 0.45 (s, 6H). ^{13}C-NMR ($CDCl_3$, 125 MHz): 165.6, 163.0, 151.0, 136.7, 128.9, 124.1, 121.5, 112.1, 109.5, 40.2, 25.6, 17.9, -3.6. MALDI-TOF (DCTB): m/z 500.30 $[M]^+$. m/z calcd for $C_{30}H_{40}N_4OSi$ 500.297 $[M]^+$. HRMS (ESI): m/z 501.3053 $[M+H]^+$. m/z calcd for $C_{30}H_{41}N_4OSi$ 501.3050 $[M+H]^+$.

a)

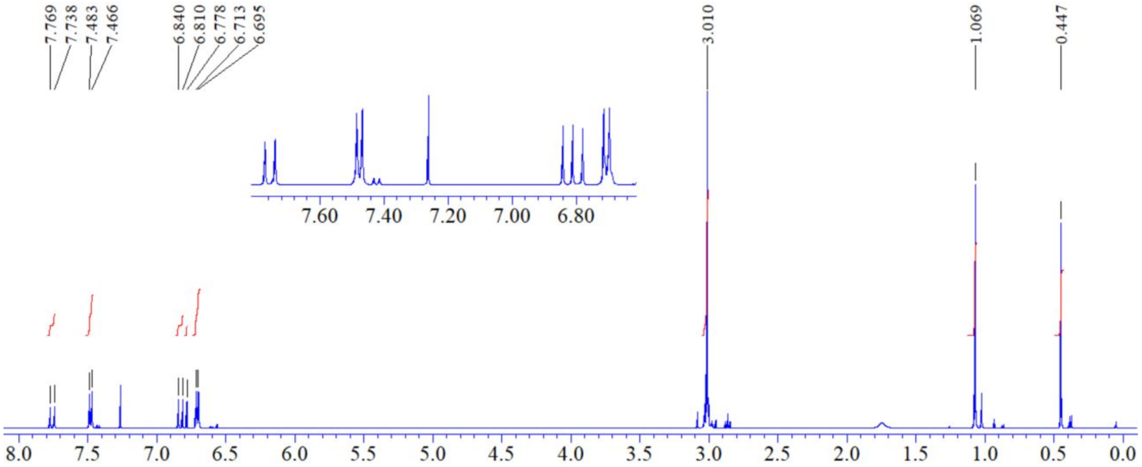

b)

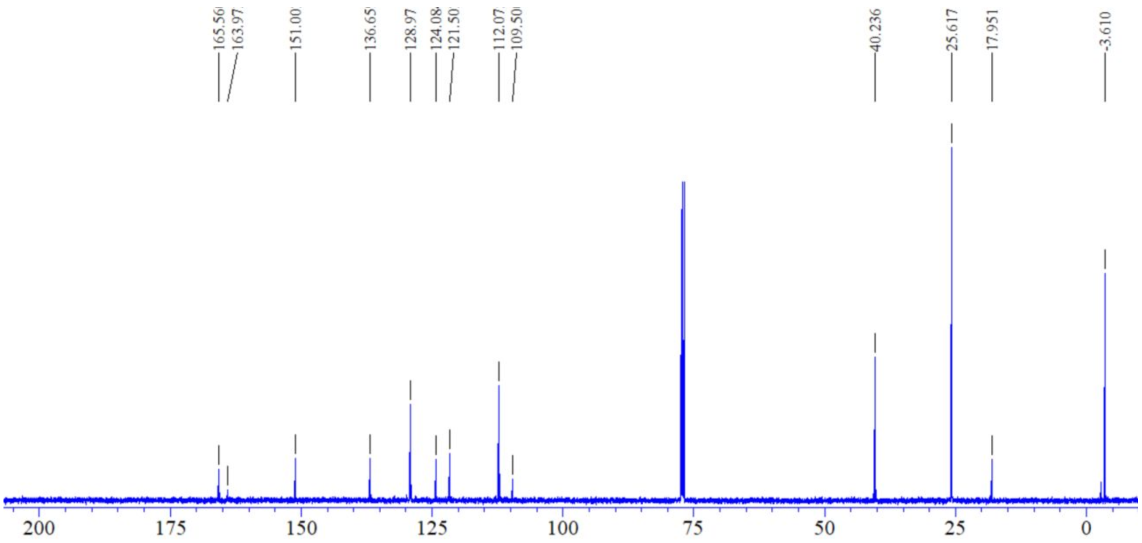

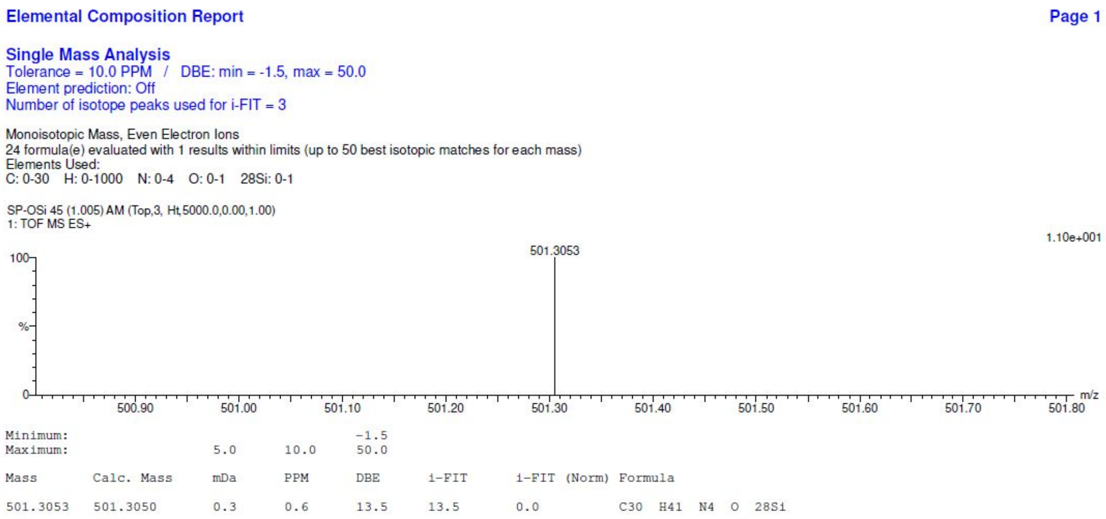

Figure S1. a) ^{1}H-NMR and b) ^{13}C-NMR spectra of **SP-OSi**. c) HRMS of **SP-OSi.**

Generation of SP-O sample for NMR: Compound **4** (30 mg, 0.078 mmol) was dissolved in THF and TBAF (78 μL of 1M solution in THF, 2.0 eq.) was added at room temperature. After 5 minutes, solvent was evaporated and the residue directly dissolved in $CDCl_3$ for the preparation of the stock solution. The NMR sample was accordingly prepared from this stock solution. . ^{1}H-NMR (400 MHz, $CDCl_3$) δ 7.61 (d, *J*= 16.0 Hz, 2H), 7.43-7.38 (m, 4H), 6.70 (d, *J*= 16 Hz, 2H), 6.67-6.59 (m, 4H), 6.15 (s, 1H), 2.95 (s, 12H). HRMS (ESI): m/z 391.2167 $[M+Li]^{+}$. m/z calcd for $C_{24}H_{24}N_4OLi$ 391.2180 $[M+Li]^{+}$.

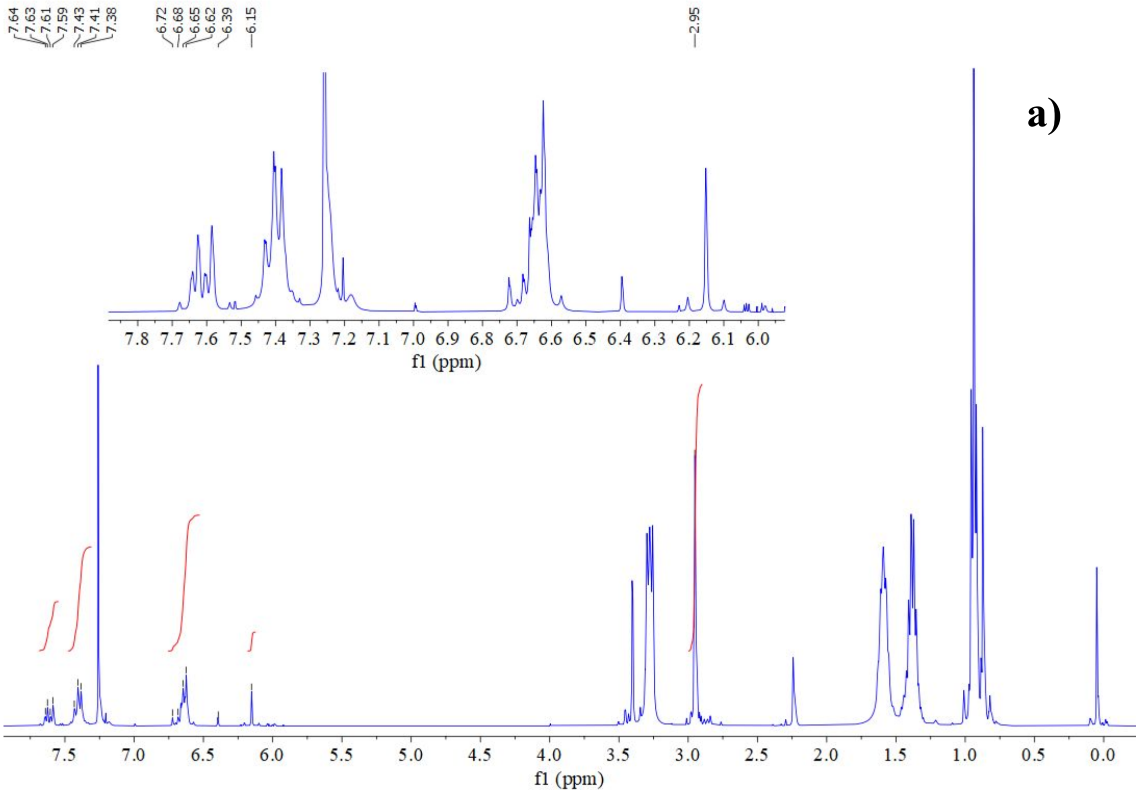

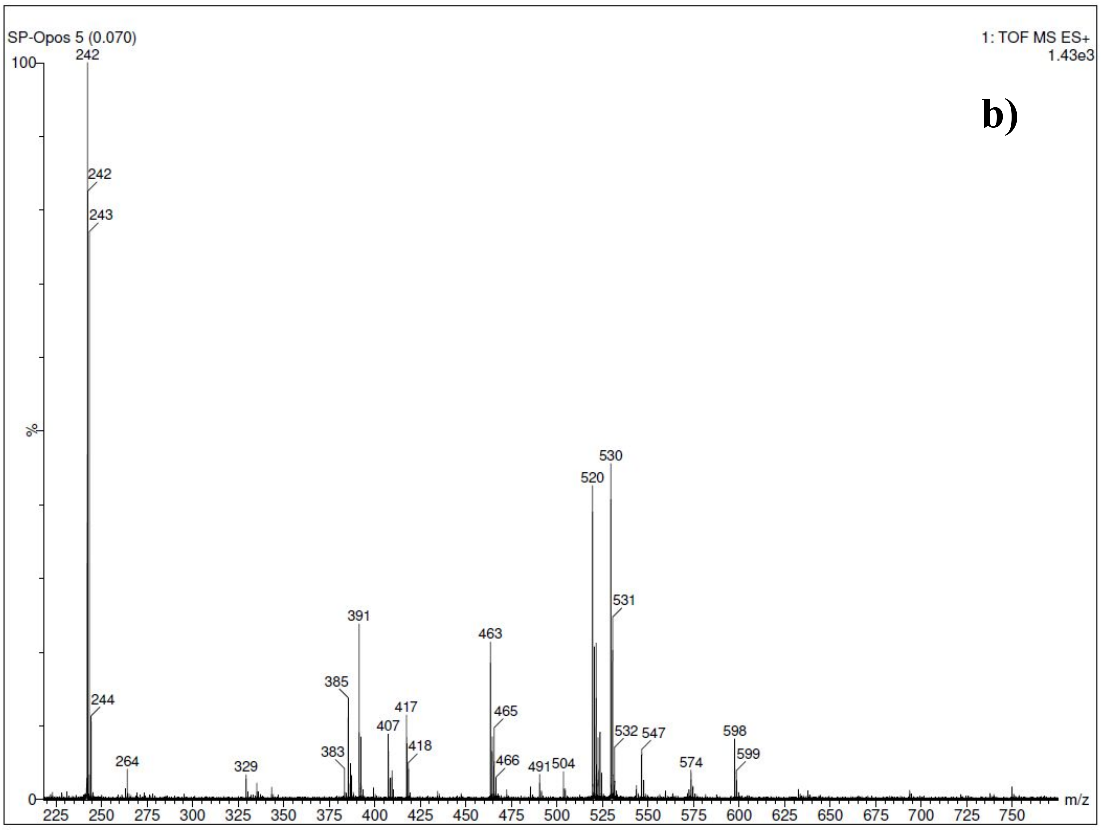

Elemental Composition Report **Page 1**

c)

Single Mass Analysis
Tolerance = 10.0 PPM / DBE: min = -1.5, max = 50.0
Element prediction: Off
Number of isotope peaks used for i-FIT = 3

Monoisotopic Mass, Odd and Even Electron Ions
102 formula(e) evaluated with 2 results within limits (up to 50 best isotopic matches for each mass)
Elements Used:
C: 0-30 H: 0-1000 6Li: 0-1 N: 0-4 O: 0-1
SP-Opos 8 (0.141) AM (Top,1, Ht,5000.0,0.00,1.00)
1: TOF MS ES+

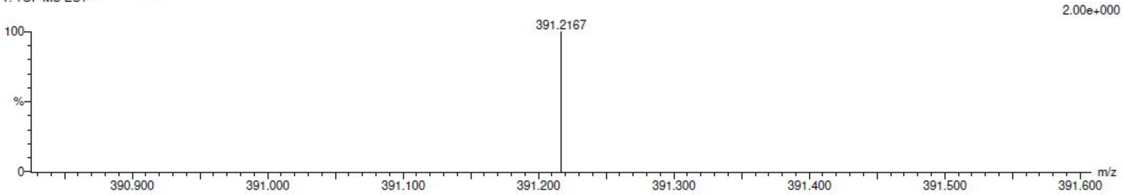

Minimum:				-1.5			
Maximum:		5.0	10.0	50.0			
Mass	Calc. Mass	mDa	PPM	DBE	i-FIT	i-FIT (Norm)	Formula
391.2167	391.2180	-1.3	-3.3	14.0	13.3	0.7	C24 H25 6Li N4 O
	391.2174	-0.7	-1.8	16.5	13.3	0.7	C28 H27 N2

Figure S2. a) ^{1}H-NMR, b) MS sweep spectrum and c) HRMS of **SP-O** $[M+Li]^+$

3. Spectroscopic Properties in Solution

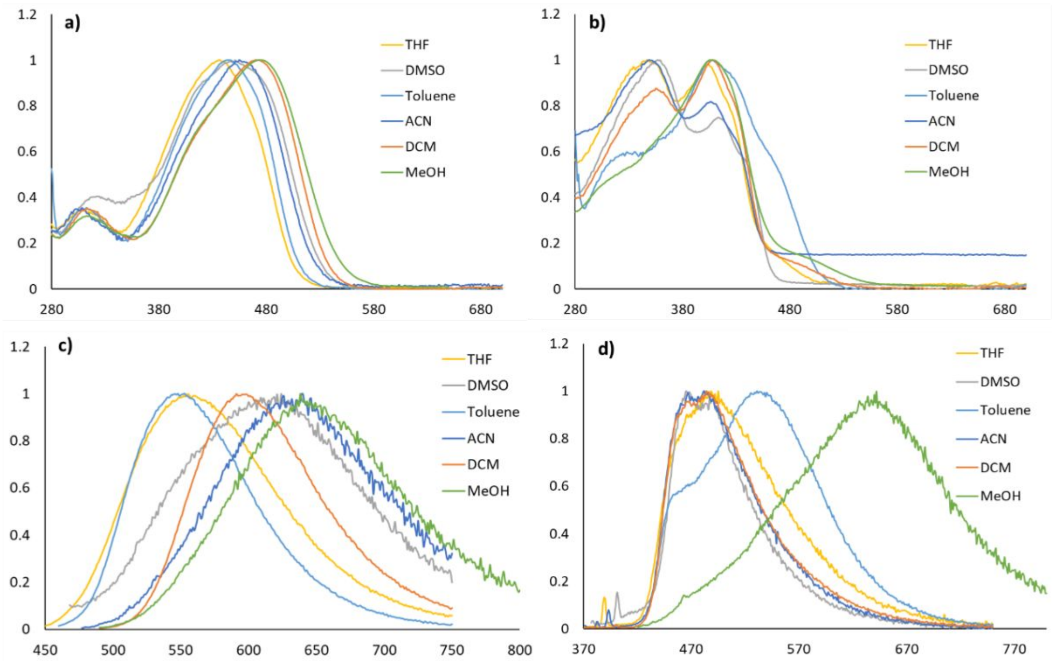

Figure S3. Absorption spectra for a) **SP-OSi** and, b) **SP-O** in different solvents. c) Emission spectra of **SP-OSi** and, d) **SP-O** in different solvents.

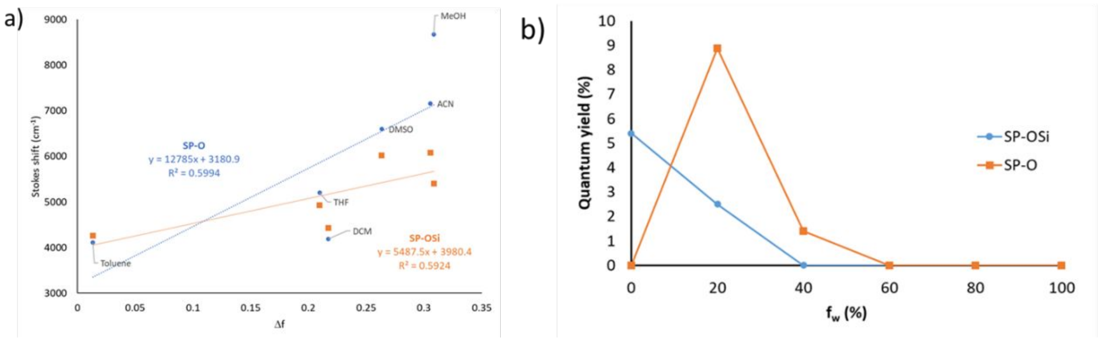

Figure S4. a) Lippert-Mataga diagram of **SP-OSi** and **SP-O**. b) Aggregation solvent-non-solvent experiment of **SP-OSi** and **SP-O** in DMSO/H_2O

4. Computational details

The molecular geometry of the ground, anionic and first excited states were optimized using the density functional theory (DFT) method implemented in the Gaussian 16 programme package (version A.03).[2]

---

[2] M. J. Frisch, G. W. Trucks, H. B. Schlegel, G. E. Scuseria, M. A. Robb, J. R. Cheeseman, G. Scalmani, V. Barone, G. A. Petersson, H. Nakatsuji, X. Li, M. Caricato, A. V. Marenich, J. Bloino, B. G. Janesko, R. Gomperts, B. Mennucci, H. P. Hratchian, J. V. Ortiz, A. F. Izmaylov, J. L. Sonnenberg, D. Williams-Young, F. Ding, F. Lipparini, F. Egidi, J. Goings, B. Peng, A. Petrone, T. Henderson, D. Ranasinghe, V. G. Zakrzewski, J. Gao, N. Rega, G. Zheng, W. Liang, M. Hada, M. Ehara, K. Toyota, R. Fukuda, J. Hasegawa, M. Ishida, T. Nakajima, Y. Honda, O. Kitao, H. Nakai, T. Vreven, K. Throssell, J. A. Montgomery, Jr., J. E. Peralta, F. Ogliaro, M. J. Bearpark, J. J. Heyd, E. N. Brothers, K. N. Kudin, V. N.

M06-2X functional was used along with the 6-31+G** basis set which has proven to be suitable to estimate the singlet excited state in previous works.[3] The vibrational frequencies were also calculated for both ground and excited stets to check the absence of imaginary frequencies. The energy barrier to rotation of the N-C-C-C dihedral angle was calculated to determine the most stable conformation (see Figure S3). The polarisable continuum model (PCM) was used to consider the effect of the solvent as implemented in the Gaussian package.[4] Time-dependent DFT calculations at the TD-M06-2X/6-31+G** were performed in dimethylsulfide. The reorganisation energy upon excitation, λ, was calculated using the programme DUSHIN developed by Reimers[5] according to:

$$\lambda = \sum_i \lambda_i = \sum_i \hbar \omega_i S_i$$

where ω_i is the wavenumber associated with the vibrational mode i and S_i is the Huang–Rhys (HR) factor.

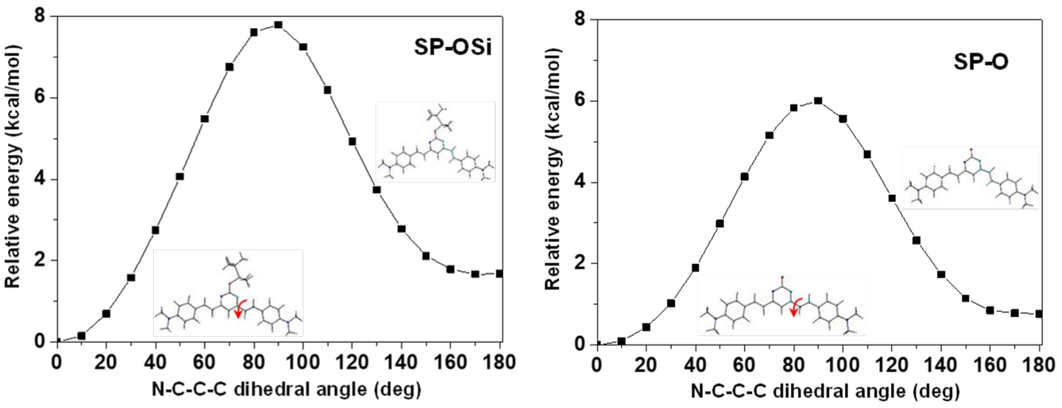

Figure S5. Energy barrier of the ground state for compounds **SP-OSi** and **SP-O** in DMSO computed at the at the M06-2X/6-31+G** level of theory.

Staroverov, T. A. Keith, R. Kobayashi, J. Normand, K. Raghavachari, A. P. Rendell, J. C. Burant, S. S. Iyengar, J. Tomasi, M. Cossi, J. M. Millam, M. Klene, C. Adamo, R. Cammi, J. W. Ochterski, R. L. Martin, K. Morokuma, O. Farkas, J. B. Foresman, and D. J. Fox, Gaussian, Inc., Wallingford CT, 2016.

[3] (a) R. Domínguez, M. Moral, M. P. Fernández-Liencres, T. Peña-Ruiz, J. Tolosa, J. Canales-Vázquez, J. C. García-Martínez, A. Navarro, A. Garzón-Ruiz, Understanding the Driving Mechanisms of Enhanced Luminescence Emission of Oligo(styryl)benzenes and Tri(styryl)-s-triazine *Chem. – A Eur. J.* **2020**, *26*, 3373–3384. (b) M. Moral, R. Domínguez, M. P. Fernández-Liencres, A. Garzón-Ruiz, J. C. García-Martínez, A. Navarro, *J. Chem. Phys.* **2019**, *150*, 64309

[4] J. Tomasi, B. Mennucci, R. Cammi, Quantum Mechanical Continuum Solvation Models, Chem. Rev. 105 (2005) 2999–3094. https://doi.org/10.1021/cr9904009. M. Cossi, N. Rega, G. Scalmani, V. Barone, Energies, structures, and electronic properties of molecules in solution with the C-PCM solvation model, J. Comput. Chem. 24 (2003) 669–681. https://doi.org/10.1002/jcc.10189.c

[5] J.R. Reimers, A practical method for the use of curvilinear coordinates in calculations of normal-mode-projected displacements and Duschinsky rotation matrices for large molecules, J. Chem. Phys. 115 (2001) 9103–9109. https://doi.org/10.1063/1.1412875.

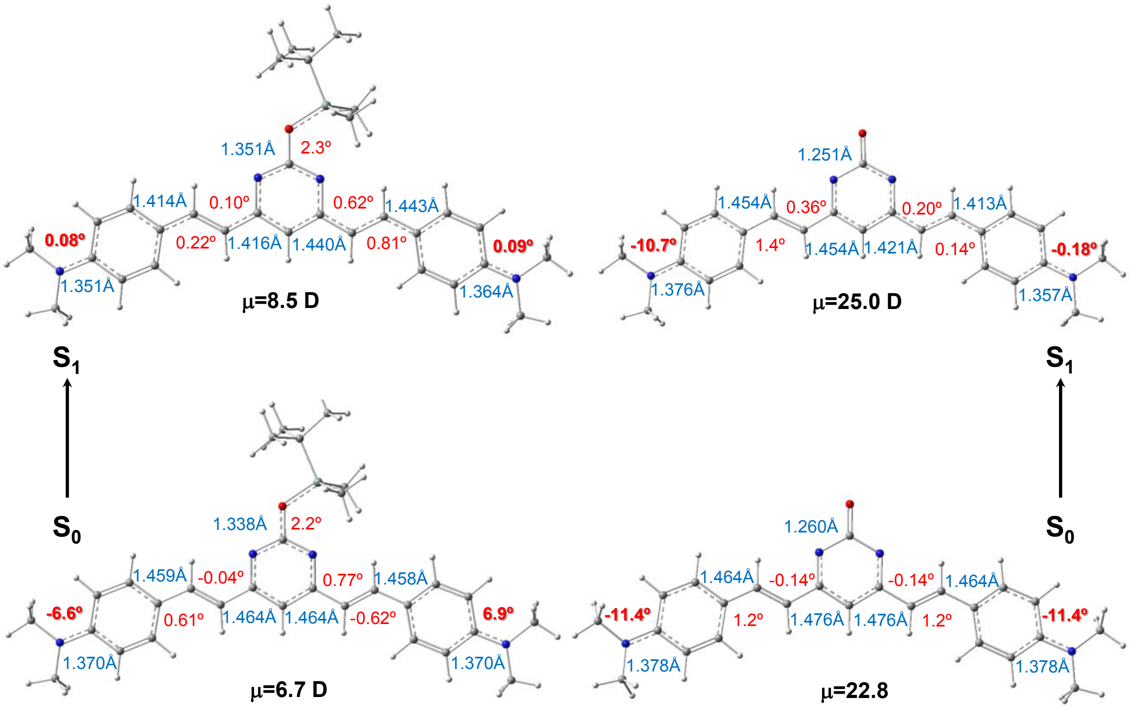

SP-OSi **SP-O**

Figure S6. Some selected geometrical parameters calculated for the S_0 and S_1 states of compounds **SP-OSi** and **SP-O** in DMSO at the M06-2X/6-31+G** level of theory. The dipole moments are included for comparison.

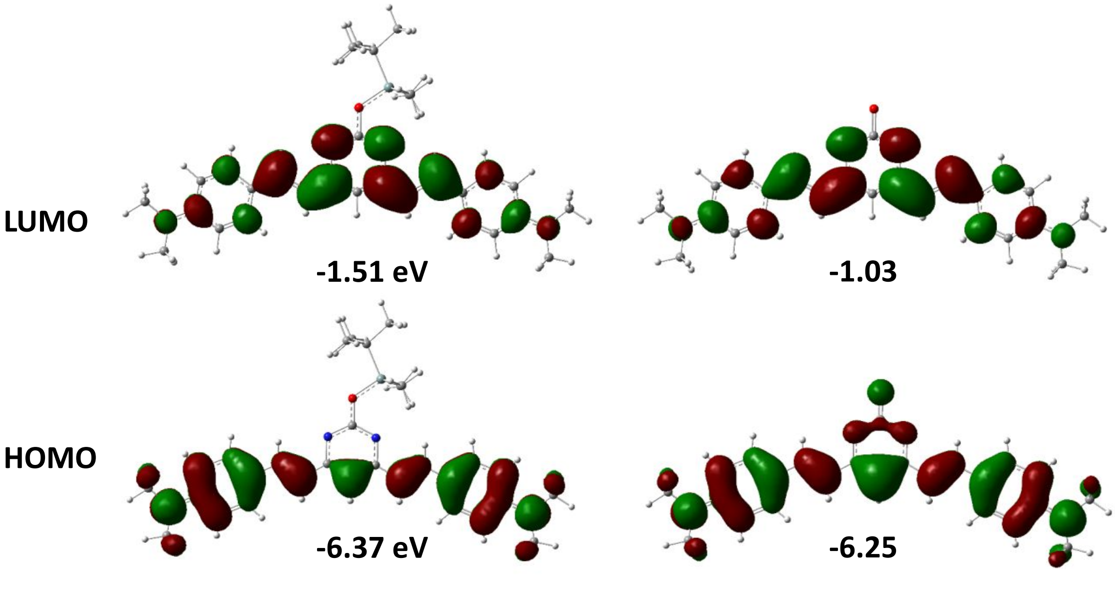

SP-OSi **SP-O**

Figure S7. Isocontour plots (0.02 au) of frontier MOs of compounds **SP-OSi** and **SP-O** in DMSO computed at the at the M06-2X/6-31+G** level of theory. The energy levels (in eV) have also included for each compound.

Table S1. Calculated lowest-energy transition wavelengths (λ_{ab}^{calc}), oscillator strengths (*f*) and orbital contributions for these transitions. Calculations were carried out at the TD-M062X/6-31+G** level of theory in DMSO solution. Calculated wavelengths (λ_{emis}^{calc}) for the $S_1 \rightarrow S_0$ transition.

Comp.	λ_{ab}^{calc} (nm [eV])	*f*	Transition	Main component of the transition (≥ 10% contribution)	λ_{emis}^{calc} (nm [eV])
**SP-OSi**	397[3.12]	2.17	$S_0 \rightarrow S_1$	H→L(86)	514[2.41]
	355[3.50]	0.23	$S_0 \rightarrow S_2$	H-1→L(85)	
**SP-O⁻**	374[3.31]	1.74	$S_0 \rightarrow S_1$	H→L(81)	506[2.45]
	331[3.75]	0.24	$S_0 \rightarrow S_2$	H-1→L(80);H→L+1(13)	
	317[3.92]	0.42	$S_0 \rightarrow S_4$	H-2→L(74); H-1→L+1(15)	

Table S2. Normal modes (ω_i in cm^{-1}) of the ground state, reorganization energy (λ_i in meV) and Huang–Rhys factors (S_i) calculated for compounds **SP-OSi** and **SP-O** in DMSO at the M06-2X/6-31+G** level of theory.

**SP-OSi**			**SP-O**		
ω_i	λ_i	S_i	ω_i	λ_i	S_i
18	3	1.34	23	5	1.75
27	1	0.30	60	1	0.13
41	1	0.20	78	1	0.10
67	1	0.12	96	4	0.34
117	1	0.07	99	5	0.41
124	1	0.06	132	1	0.06
198	1	0.04	158	1	0.05
255	1	0.03	271	1	0.03
538	2	0.03	421	1	0.02
539	1	0.01	441	1	0.02
764	2	0.02	539	3	0.04
878	2	0.02	611	1	0.01
880	2	0.02	714	1	0.01
1019	8	0.06	759	2	0.02
1020	1	0.01	871	2	0.02
1021	3	0.02	983	5	0.04
1140	1	0.01	1138	1	0.01
1141	2	0.01	1142	1	0.01
1145	4	0.03	1144	1	0.01
1162	1	0.01	1149	1	0.01
1173	3	0.02	1200	1	0.01
1202	1	0.01	1209	4	0.03
1207	6	0.04	1210	9	0.06
1210	5	0.03	1227	2	0.01
1253	1	0.01	1248	1	0.01
1275	8	0.05	1265	16	0.10
1336	5	0.03	1331	7	0.04

1346	5	0.03	1336	4	0.02
1354	3	0.02	1350	2	0.01
1379	1	0.01	1372	2	0.01
1408	8	0.05	1397	7	0.04
1410	2	0.01	1402	1	0.01
1427	1	0.01	1410	2	0.01
1482	1	0.01	1447	1	0.01
1482	3	0.02	1477	3	0.02
1501	1	0.01	1566	4	0.02
1517	1	0.01	1575	1	0.01
1532	1	0.01	1580	1	0.01
1576	12	0.06	1587	4	0.02
1597	45	0.23	1612	33	0.16
1620	1	0.00	1625	1	0.00
1626	8	0.04	1681	13	0.06
1678	27	0.13	1682	18	0.09
1712	4	0.02	1723	19	0.09
1726	31	0.14	1734	55	0.26

5. Sensor in Solution

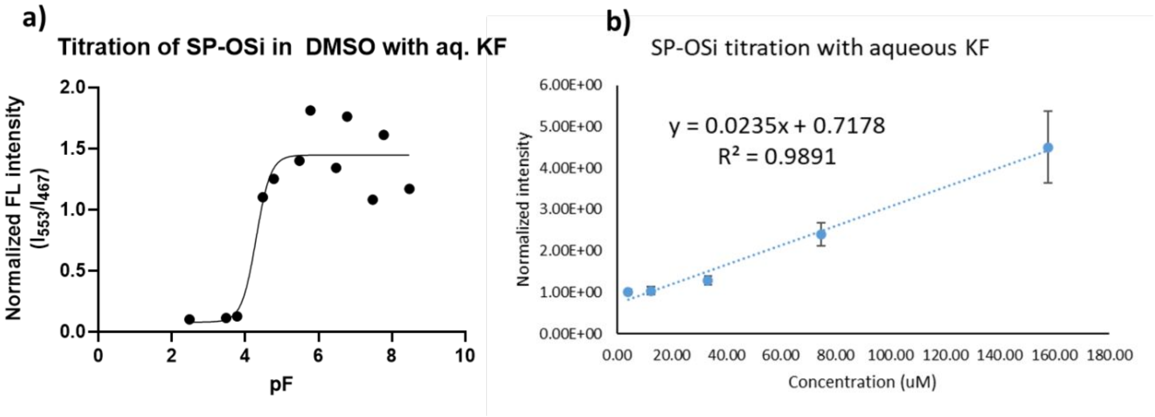

Figure S8. (a) Titration of **SP-OSi** with increasing concentrations of fluoride ions, (b) calibration curve.

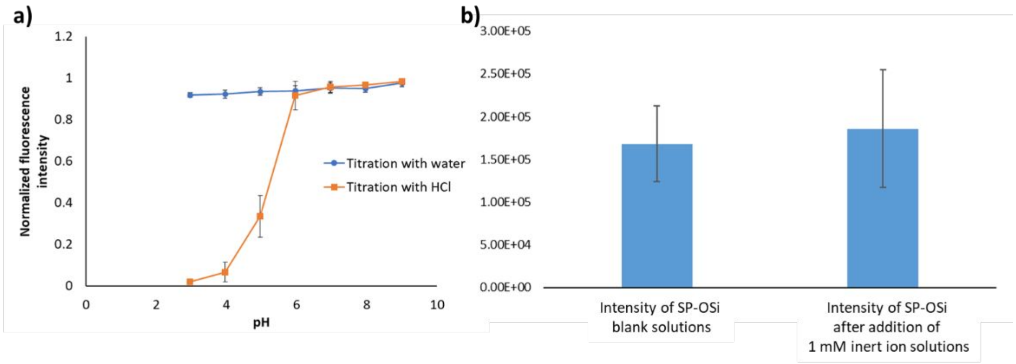

Figure S9. Effect of acidity and polarity on SP-OSi solutions in DMSO. (a) Titration of **SP-OSi** with increasing concentrations of water and HCl. (b) Comparison of the intensity of SP-OSi in DMSO before and after the addition of different inert ions solutions.

Figure S9 shows the titration of SP-OSi with acid, exhibiting a single equivalence point at pH=5.18. When a similar titration was conducted using an equivalent amount of water to the case of HCl, the quenching of fluorescence was negligible even after adding 7 times the volume usually employed for titrations (2.5 µL). This experiment supports the fact that variation of fluorescence is not caused by the increase of polarity due to the addition of water. To study any effect of a polarity, increase due to the presence of ions, the averages of emission intensities for SP-OSi solutions in DMSO before and after the addition (2.5 µL) of different inert salts at 1 mM concentrations were compared. Figure S6b shows the average values and their standard deviations (n = 18). These averages were compared using a two-tailed t-test assay with Welch's correction at a 95% confidence level; obtained p-value was 0.3778, which stated that there were no significant differences between them, pointing to the absence of any polarity influence on the fluorescence using the reported volumes of aqueous solutions.

Method	Fluorescence
Measurement range, µM	4.2 – 157.7
Slope	0.024
Intercept	0.72
LOD, µM	14.5
LOQ, µM	48.3
Precision, %	1.5

Table S4. Analytical parameters of µPAD for fluoride ions determination.

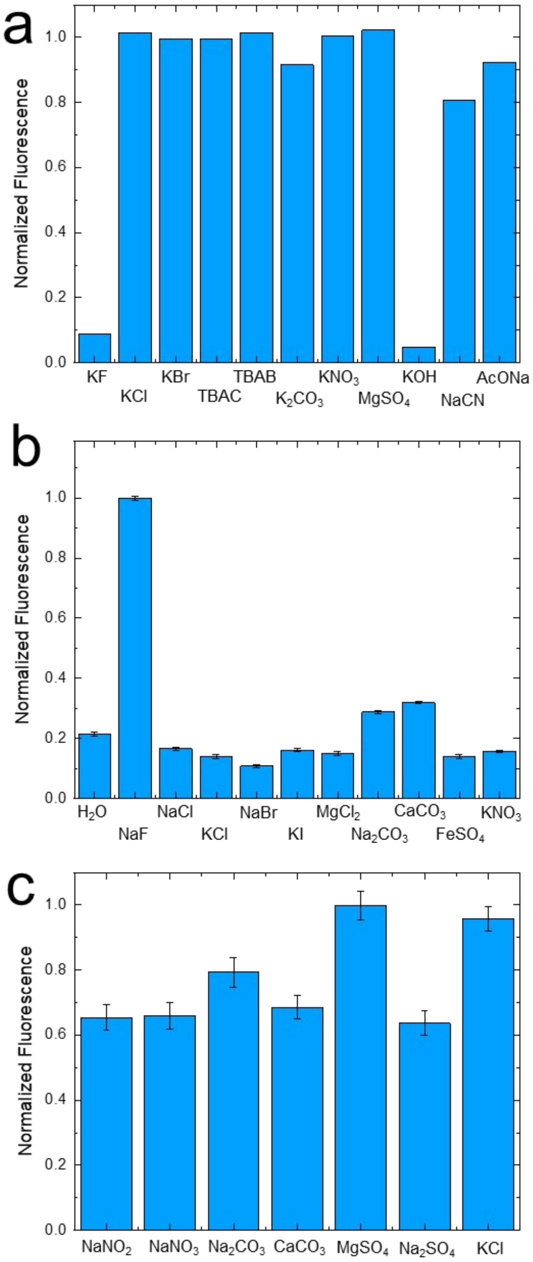

Figure S10. Selectivity of **SP-OSi** towards different anions in (a) DMSO solution and in (b) μPAD system. (c) Fluorescence of μPAD after incubation with mixtures of 20 μM NaF in the presence of 20 μM of different ions

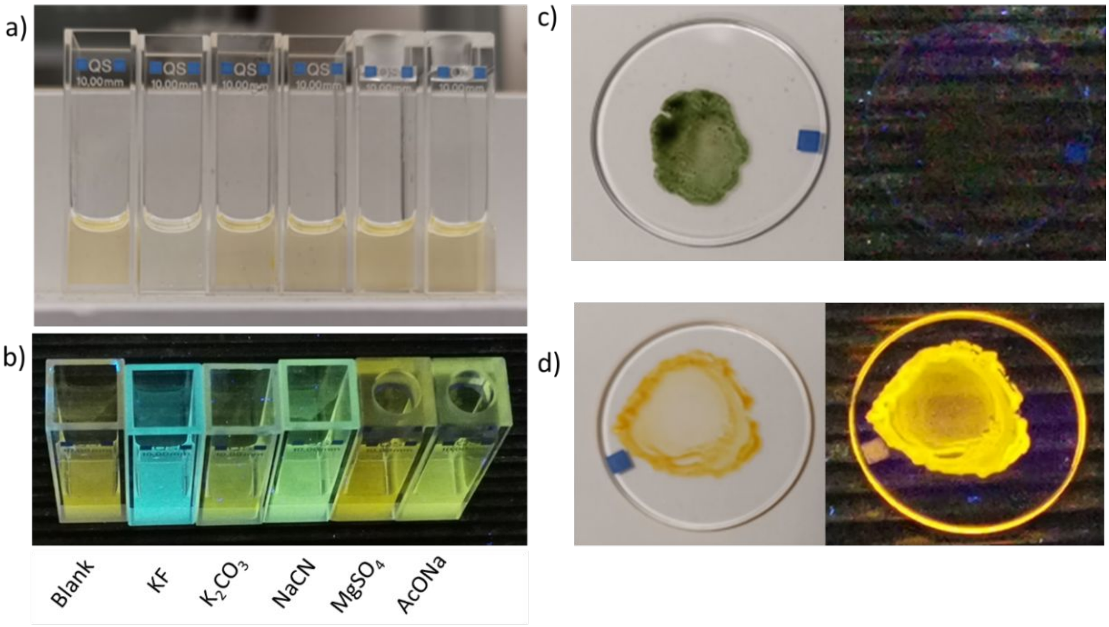

Figure S11. Pictures of solutions of **SP-OSi** in DMSO in, a) visible light and b) under UV lamp irradiation (365 nm) after the addition of several different salts as aqueous solutions. Pictures of dropcast samples of c) **SP-OSi** and d) **SP-O** in visible light and under UV lamp irradiation (365 nm).

6. Fabrication and preparation of the µPAD

Microfluidic paper-based analytical devices were fabricated using a paper sheet (Whatman nº1) by craft-cutting employed a CO_2 laser engraver. The pattern of the µPAD was first designed using Illustrator software (Adobe Systems) and the design was cut by CO_2 laser.

The µPAD consists of two layers prepared individually, as shown in Figure S7. The first layer is the detection area where the **SP-OSi** is immobilized. The second layer is made up of two separates areas, one for sampling and the second for transport of the sample. To prepare the µPAD, 4 µL of **SP-OSi** in 10 µM THF solution was dispensed onto the detection area by drop casting and 2 µL of phosphate buffer 100 mM pH 7 was added on the transport channel, both pieces of paper were dried at room temperature for 2 min and stored in a dry environment at 4ºC in the dark.

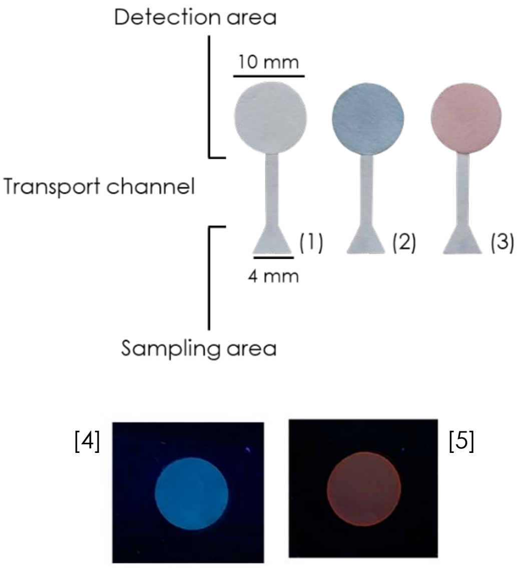

Figure S12. (1) Picture of the μPAD device showing the two layers: detection area, transport channel and sampling area. μPAD after reaction of the sample (2) without and (3) with fluoride sample. Additionally, the μPAD under UV irradiation (4) without and (5) with fluoride sample are shown.

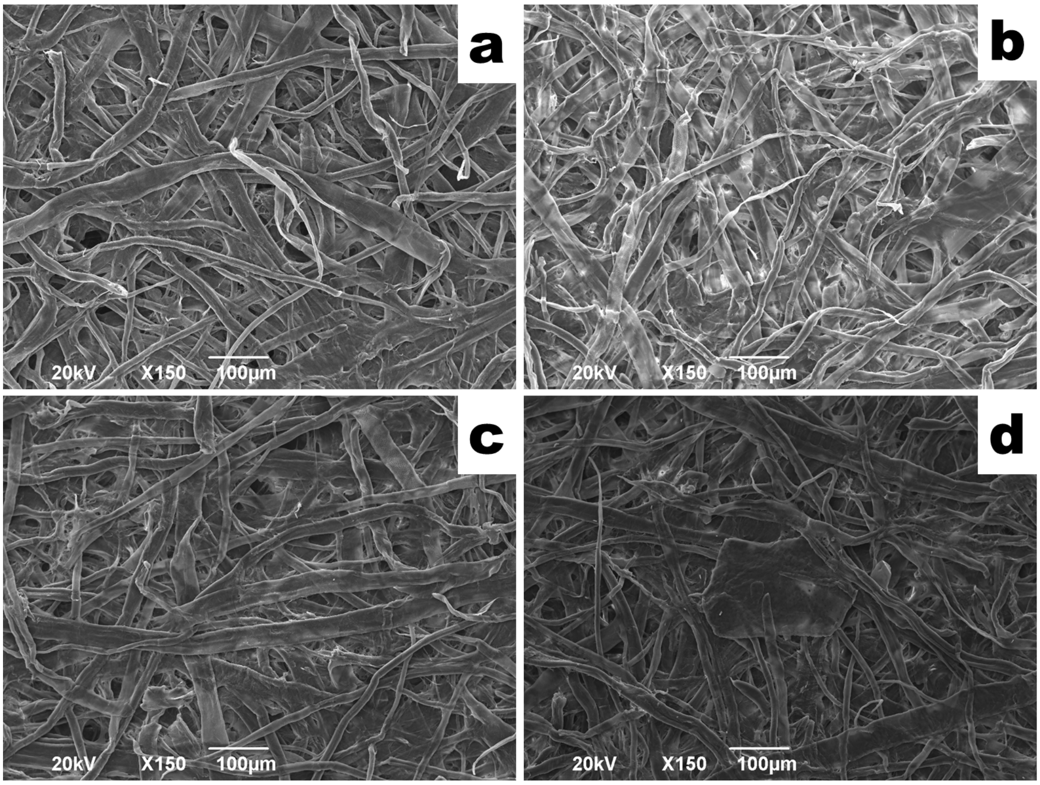

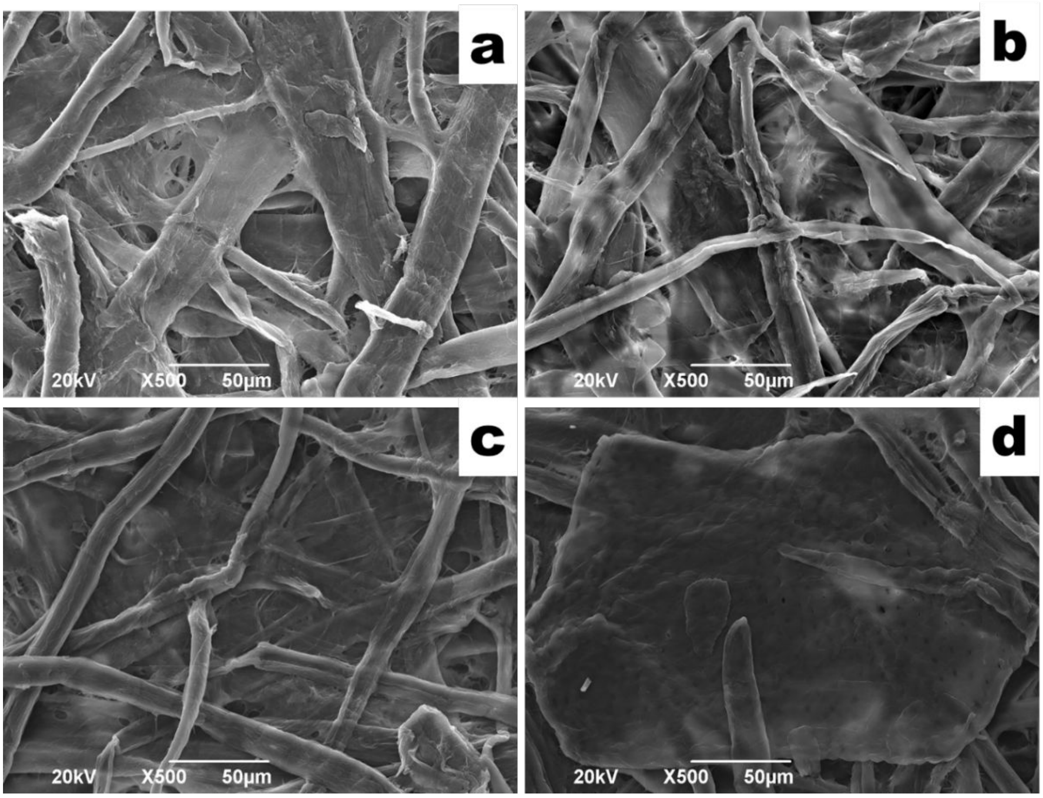

Figure S13. SEM of μPAD samples. a) Whatmann 1 paper untreated, b) μPAD soaked with **SP-OSi**, c) μPAD soaked with **SP-OSi** after fluoride ion determination, and d) μPAD soaked with **SP-OSi** in buffered solution after fluoride ion determination.

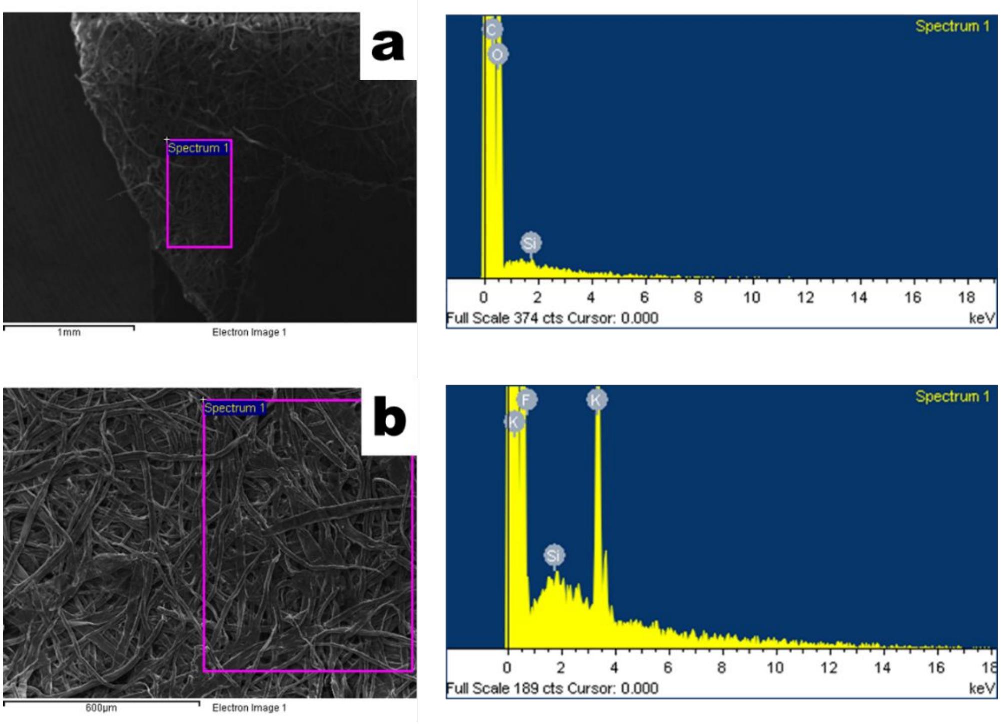

Figure S14. EDS of a) μPAD soaked with **SP-OSi** and b) μPAD soaked with **SP-OSi** after fluoride ion determination.

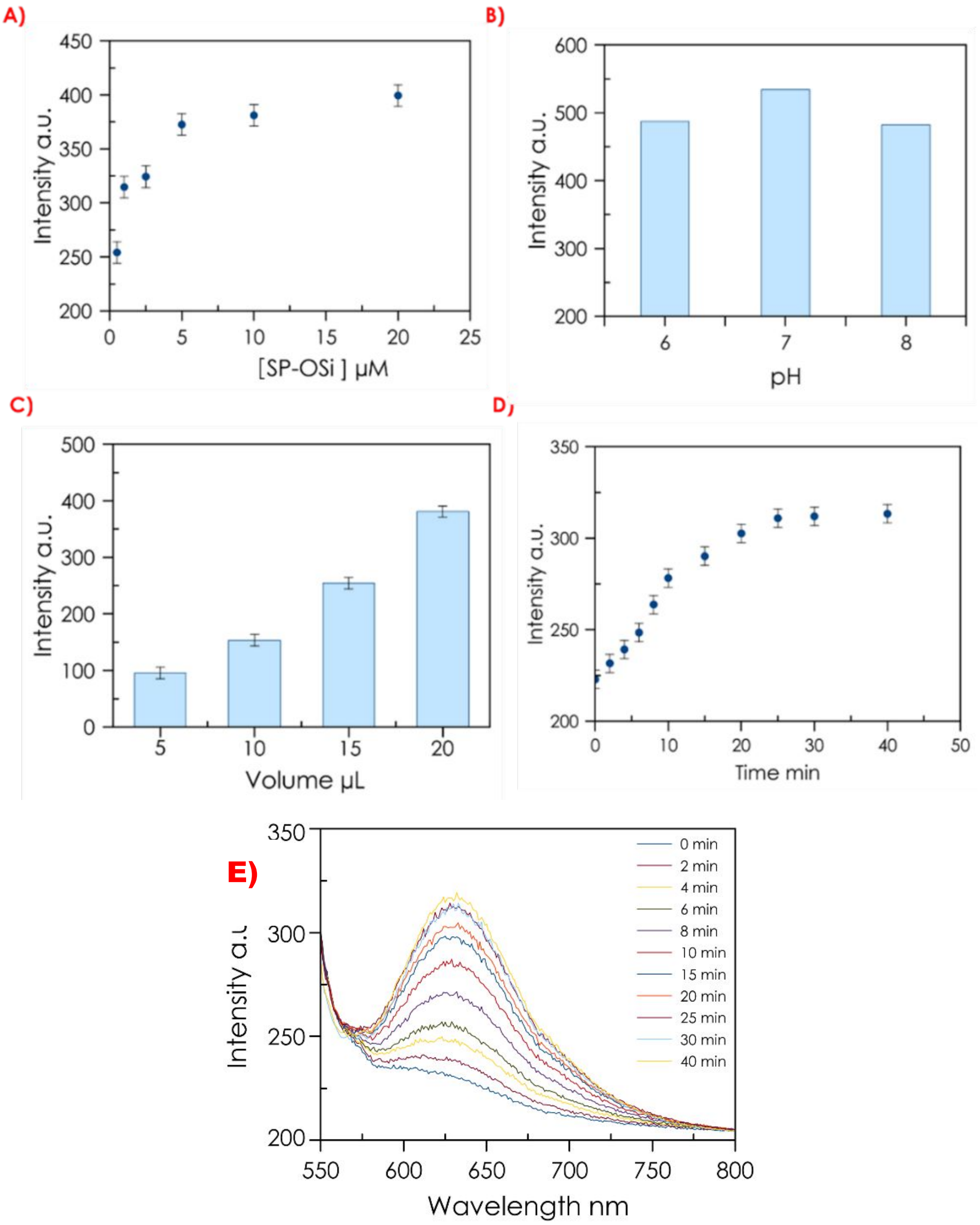

Figure S15. Influencing factors for fluoride determination in the developed μPAD. (a) concentration of **SP-OSi**; (b) pH; (c) volume of sample and (d) reaction time. e) time-dependent fluorescence spectra of the SP-OSi reacting with fluoride.

SP-OSi exhibits almost no emission when excited at 530 nm and no distinct variations are observed in the assay condition, suggesting that **SP-OSi** is stable and not converted into **SP-O**$^-$. However, the fluorescence intensity of **SP-OSi** increased when fluoride ions was added to the device. A significant turn-on fluorescence response at 617 nm was observed. Furthermore, the fluorescence intensities at 617 nm have a linear relationship (R^2=0.994) within the fluoride ions

concentrations from 11.9 – 125.0 µM, and the detection limit was evaluated to be 3.9 µM, indicating that **SP-OSi** has a very good sensitivity to the detection of fluoride.

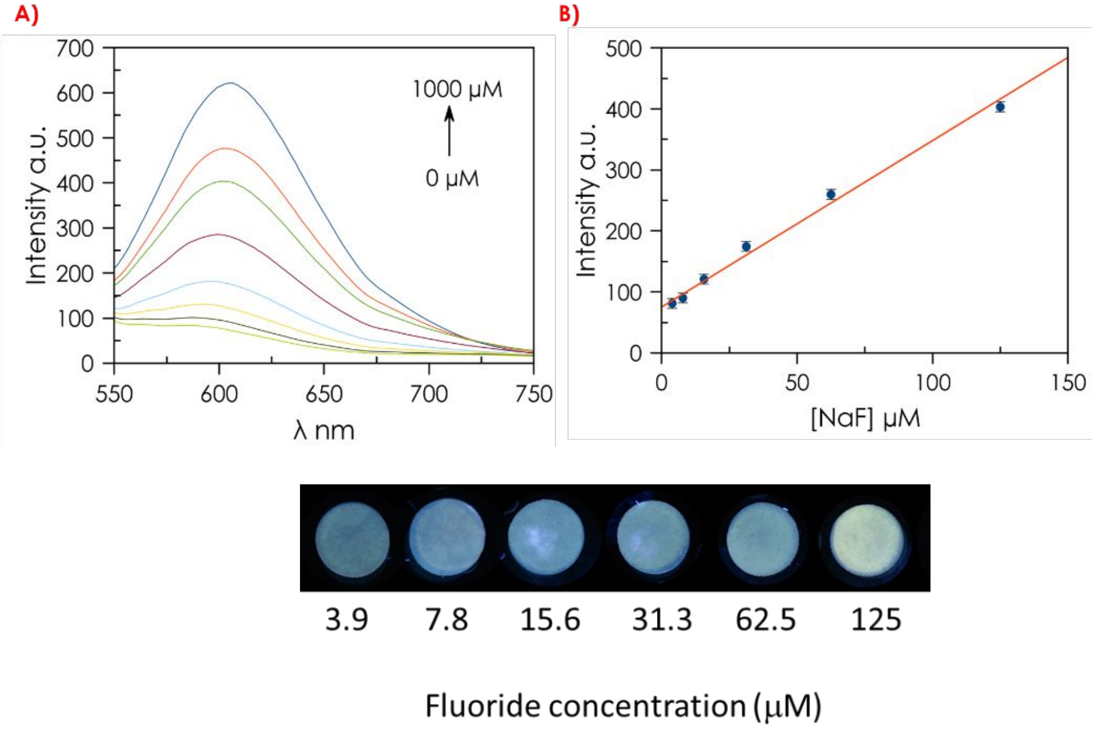

Figure S16. (a) Emission spectral response of **SP-OSi** increasing concentrations of fluoride ions, (b) calibration curve and c) picture of µPADs used to build the calibration curve.

Method	Fluorescence
Measurement range, µM	11.9 – 125.0
Slope	3.5
Intercept	75.9
LOD, µM	3.9
LOQ, µM	11.9
Precision, %	1.3

Table S5. Analytical parameters of µPAD for fluoride ions determination.

7. Treatment of real samples

In order to evaluate the operating applicability of the sensor in real samples it was carried out the analysis of samples of tap water, brick green tea infusion and commercial mouthwash. All the samples were collected in clean polyethylene bottle and were stored at -18ºC until they were analyzed. The sample of tap water and the commercial green tea were filtered using a filter of 0.45 μm to remove the impurities. Different concentrations of fluoride ions were added to the samples of water and green tea, then these samples were analyzed using the sensor. For the sample of the mouthwash solution, a dilution of 1:100 with distilled water was carried out and the fluoride ions concentration was determined using the μPAD sensor such as the analytical protocol indicated. On the other hand, the sample of toothpaste (1g) was dissolved in 100 mL of distilled water heating for 10 minutes in a water bath. Later, 100 μL of propanol was added to reduce foaming and the sample was diluted 100-fold.[6] The concentration average of fluoride for each sample was obtained from three independent measurements and presented with the associated standard deviation.

For fluoride determination, 20 μL of a solution of different concentrations of fluoride is dropped onto the sampling area of the μPAD. After the solution flows by capillarity towards the areas where the reagents are placed, twenty minutes are needed to complete the reaction, when a blue or red colour appears in the white detection area, depends on the concentration of fluoride in the sample. Once the sensing area is allowed to react a fluorescence measurement is carried out using a luminescence spectrometer. For the luminescence measurement, the detection area of the μPAD was excited at 530 nm.

---

[6] Švarc-Gajića, J.; Stojanovića, Z.; Vasiljevićb, I; Kecojevićb I. Determination of fluorides in pharmaceutical products for oral hygiene *J. Food Drug Anal* **2013**, *21*, 384-389
